# Don’t walk on by: an innovative high risk foot clinic model

**DOI:** 10.1186/1757-1146-4-S1-P23

**Published:** 2011-05-20

**Authors:** Mark Fulton, Sarah E Brown

**Affiliations:** 1 Chronic and Complex Care, Health Independence Program, Ambulatory and Community Care, Southern Health, Springvale, Victoria, 3171, Australia

## 

Non-attendance at scheduled appointments is one of the most pressing issues in chronic illness management. Non-attendance is often influenced by personal and clinical factors and issues pertaining to the nature and operations of the clinic (the way health services are structured, organized and delivered affect access and satisfaction with services that in turn affect patients’ ability and motivation to attend chronic disease clinics). Accessibility can be improved through flexible hours of operation, effective engagement and alternative ways of providing education and delivering services. A “drop-in” clinic operates at the Springvale site of Southern Health’s Greater Dandenong Community Health Service. It provides evidence based prevention and management of diabetes related foot complications including: (i) prevention of foot ulcers through education, early screening and case management; (ii) early intervention of ulcers to optimise healing, prevent infections and hospital admissions; (iii) links with Southern Health’s High Risk Foot Clinic and other ambulatory clinics; (iv) management planning for foot complications that are not due to ulcer, soft tissue or osseus complaint. The clinic’s scope of practice is confined to high risk foot: foot wounds, diabetic assessment, complicated foot complaints, psycho-social foot complaints, foot-wear and foot-gear assessment. Living Well, the broader program within which the clinic operates, provides support to assist clients experiencing homelessness (primary, secondary or tertiary) to manage diabetes, musculoskeletal or respiratory conditions and cardiovascular disease. Typically, clients experience a combination of mental illness, intellectual disability, acquired brain injury, chronic physical health problems, behavioural difficulties and drug and/or alcohol use. Clients are engaged via assertive outreach activities, in-reach into pension-level supported residential services, discharged from acute services (including acute-based high risk foot clinics) or are existing clients of Living Well. An inter-disciplinary approach with diabetes nurse, key worker (care coordinator) and dietician ensures that needs “beyond the foot” can be addressed. A Community Health Nurse from Royal District Nursing Homeless Person’s Program is also contracted to provide services to the program’s clients. Clients are supported to attend health appointments and access foot-wear and other items (for example, medications) which are integral to the client’s wellbeing.

